# When communities collide

**DOI:** 10.7554/eLife.18753

**Published:** 2016-07-15

**Authors:** Jason Merritt, Seppe Kuehn

**Affiliations:** Department of Physics, University of Illinois at Urbana-Champaign, Urbana, United States; Department of Physics, University of Illinois at Urbana-Champaign, Urbana, United Statesseppe@illinois.edu

**Keywords:** microbial ecology, consortia, cooperation, resource competition, niche construction, None

## Abstract

A new model demonstrates how microbial communities can survive encounters with other communities as a cohesive group, even in the complete absence of cooperation.

**Related research article** Tikhonov M. 2016. Community-level cohesion without cooperation. *eLife* **5**:e15747. doi: 10.7554/eLife.15747

**Image** The overall fitness of a microbial community predicts its ability to compete for resources

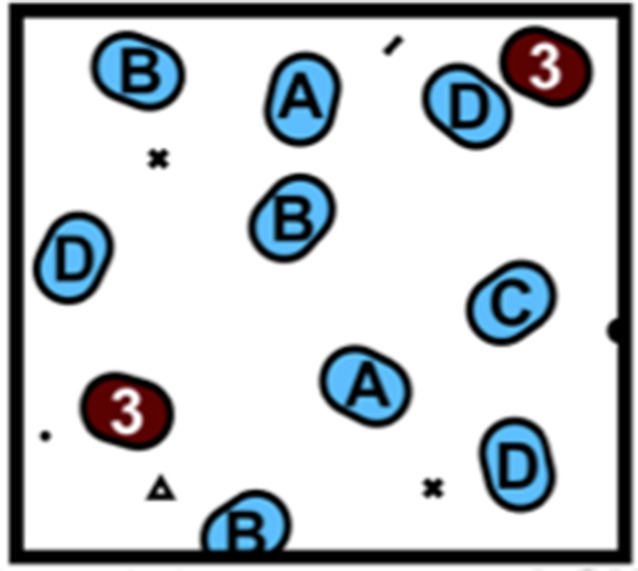

In an unending process that Charles Darwin called “the struggle for existence,” all organisms compete for survival. Studies of microbes, which live in complex communities that contain hundreds of interacting species, have taught us much about the dynamics of this struggle (Vetsigian et al., 2011). Moreover, we have recently learned that entire microbial communities can behave as cohesive structures, responding to change and challenge as if each community was a single organism. In the case of fecal transplants, for example, when a healthy microbial community is introduced into the bowel of a sick patient, we often observe the new community outcompeting the resident community responsible for the disease (Khoruts et al., 2010).

But why and how does one of these microbial communities outcompete another? What makes them behave in a cohesive way, instead of collapsing into their constituent species under the pressure of such competition? Now, in eLife, Mikhail Tikhonov of Harvard University reports how a simple modeling framework can help us to understand what happens when microbial communities collide (Tikhonov, 2016).

One way to understand how one microbial community competes with another is to take a “bottom-up” approach and describe the interactions between each species in the community. Within a single community, however, many chemical, physical and ecological processes happen simultaneously. Given the sheer number of processes at work when two communities compete, predicting the outcome of a competition event from the bottom up is daunting. Even so, sophisticated computational models may provide a route toward making such predictions (Klitgord and Segrè, 2010; Freilich et al., 2011).

Another way to explore the mysteries of this competition is to look at the problem from a “top-down” perspective. Here the idea is to use simplified mathematical models that focus on large-scale changes to look for statistical or organizational principles that shape communities (O'Dwyer et al., 2012; Hekstra and Leibler, 2012).

Tikhonov takes a top-down approach and builds on a classic ecological model proposed by the late Robert MacArthur almost 50 years ago (MacArthur, 1969). In the model, environments are uniform and contain multiple resources. The species that populate these environments are distinguished only by the resources they can use and by the price they pay for using those resources. Each species may consume one or many resources, although consuming more resources comes at an increasing cost. Critically, the model permits no cooperation between species: they can only compete with each other for resources.

When an environment is first populated by a random set of organisms in Tikhonov’s model, the population achieves an equilibrium that is determined by the different species present in the community. You might expect that the species with the lowest nutrient costs for reproduction would come to dominate the community at equilibrium. But because the growth of each species depends on the other species present in the community, things turn out differently. For example, a species that can grow efficiently on a resource for which there is high demand from other species will ultimately be forced to share that resource, thus slowing its growth. However, a specialized species that can grow and reproduce slowly on a little-used resource will eventually flourish.

Tikhonov’s model shows that the community dynamics modeled in this way do not optimize the fitness of individuals, but instead optimize the community “fitness”, as measured by the ability of the entire population to consume resources fully: the more thoroughly a community exhausts the available resources, the more fit it is (Figure 1). A similar idea has been proposed previously for predicting the outcome of competition between species (Tilman, 1982).

**Figure 1.**
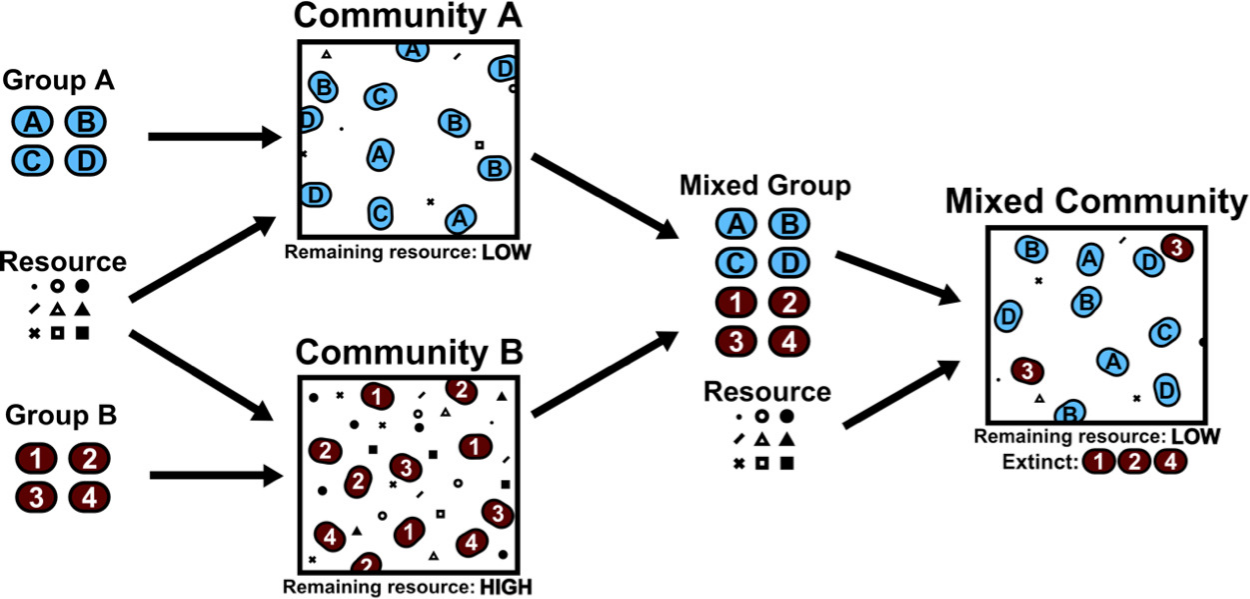
Community collision and cohesion. Two groups of randomly chosen species are separately grown in environments with various resources, yielding two distinct communities. A "high fitness" community (A, blue) consumes nearly all available resources, and a "low fitness" community (B, red) uses less of the available resources. Note that fitness is defined by the ability to consume resources; therefore otherwise low-performing individuals can form a high-fitness community as long as they consume all the resources in the environment. When species from both groups are grown together in a new environment, species from the low fitness community are more likely to go extinct, resulting in the cohesion of the high fitness community even when the interactions between species in the high-fitness community are purely competitive.

With this framework in place, Tikhonov carried out simulations in which two initial populations of different species are seeded in entirely separate environments and allowed to evolve to equilibrium. When that is achieved, the species from both environments are mixed together and allowed to equilibrate once more, and the resulting composition of the combined community is examined.

Unexpectedly, the fitness of individual species does not reliably predict which species survive and thrive during this coalescence process. Instead, the overall fitness of each initial community is a much better predictor of the composition of the final combined community. For example, a community composed of low-performing species that exhaustively depletes resources in its environment outcompetes a community of high-performing species that uses resources poorly. Tikhonov shows that this is a direct consequence of interactions that cause organisms to influence their environment because this alters the fitness of individual species regardless of how well they perform in a random environment. For example, if a community contains a species that drives a specific resource to very low levels, the presence of this species `constructs’ an environment for other species in the community where this resource level is low. Thus, remarkably, communities cohere even when all the species in the community selfishly compete with each other. At present it is not known if the mechanism proposed by Tikhonov for community cohesion is at play in real-world processes like fecal transplants, but this is an important avenue for future work.

Microbial ecologists have long used the metaphor of the community as an individual (Shapiro, 1998). Tikhonov’s model makes this metaphor mathematically exact. In particular, the changing abundances of different species in the community act in the same way as regulated metabolic pathways act within a single organism as it responds to changes in the availability of resources.

Though conventional logic might lead us to assume that cohesive communities arise from cooperative interactions, Tikhonov’s model forces us to think again, reminding us of the critical role that theory can play in helping us understand systems as complex as microbial communities. In the future, with sequencing data now available on microbial communities in virtually any setting, our search for the signatures of community cohesion during competition will be guided by theory.
